# Data-driven characterization of molecular phenotypes across heterogeneous sample collections

**DOI:** 10.1093/nar/gkz281

**Published:** 2019-04-24

**Authors:** Juha Mehtonen, Petri Pölönen, Sergei Häyrynen, Olli Dufva, Jake Lin, Thomas Liuksiala, Kirsi Granberg, Olli Lohi, Ville Hautamäki, Matti Nykter, Merja Heinäniemi

**Affiliations:** 1 Institute of Biomedicine, School of Medicine, University of Eastern Finland, Kuopio, Finland; 2 Faculty of Medicine and Health Technology, Tampere University, Tampere, Finland; 3 Hematology Research Unit Helsinki, University of Helsinki and Department of Hematology, Helsinki University Hospital Comprehensive Cancer Center, Helsinki, Finland; 4 Tampere Center for Child Health Research, Tampere University and Tampere University Hospital, Tampere, Finland; 5 School of Computing, University of Eastern Finland, Joensuu, Finland

## Abstract

Existing large gene expression data repositories hold enormous potential to elucidate disease mechanisms, characterize changes in cellular pathways, and to stratify patients based on molecular profiles. To achieve this goal, integrative resources and tools are needed that allow comparison of results across datasets and data types. We propose an intuitive approach for data-driven stratifications of molecular profiles and benchmark our methodology using the dimensionality reduction algorithm t-distributed stochastic neighbor embedding (t-SNE) with multi-study and multi-platform data on hematological malignancies. Our approach enables assessing the contribution of biological versus technical variation to sample clustering, direct incorporation of additional datasets to the same low dimensional representation, comparison of molecular disease subtypes identified from separate t-SNE representations, and characterization of the obtained clusters based on pathway databases and additional data. In this manner, we performed an integrative analysis across multi-omics acute myeloid leukemia studies. Our approach indicated new molecular subtypes with differential survival and drug responsiveness among samples lacking fusion genes, including a novel myelodysplastic syndrome-like cluster and a cluster characterized with *CEBPA* mutations and differential activity of the S-adenosylmethionine-dependent DNA methylation pathway. In summary, integration across multiple studies can help to identify novel molecular disease subtypes and generate insight into disease biology.

## INTRODUCTION

Gene expression profiling represents the most common genome-wide method for studying cells in healthy and diseased states. As a result, large repositories for data sharing across studies exist (1–3). However, in practice a major limitation to utilize these results in integrative analysis is that technical differences, rather than separation based on biological subtypes, tend to overshadow the comparison of sample molecular characteristics (4,5). In particular, this poses a challenge to characterize rare disease or rare subtypes, as combining several datasets would be necessary to obtain sufficient sample size for further analyses.

One common approach to discover different cellular states and disease types based on gene expression is to use unsupervised methods that require no prior knowledge on sample groups within a dataset (6). Dimensionality reduction (7) and clustering represent data-driven methods that are well-suited for discovery of sample grouping from complex high-dimensional gene expression data and that are typically used in combination, to mitigate the effect of the curse of dimensionality (8). In context of data generated by multiple laboratories, the contribution of technical variation to the obtained sample grouping remains a challenge (4). Moreover, including additional studies and data types into the analysis is important to evaluate robustness of the analysis and to gain deeper insight into the biological mechanisms that underlie the distinct gene expression clusters. Therefore, it would be desirable that new data can be interpreted in context of already carefully analyzed results. Yet, adding new samples to unsupervised analysis typically suffers from the limitation that the lower dimensional visualization and clustering will become different once more samples are included. One approach to overcome this limitation is to identify gene sets that define specific clusters or signatures discovered in the original analysis (9). However, this scoring is typically calculated for a set of samples as a relative measure of gene set activity (10).

Acute myeloid leukemia (AML) multi-omics profiling (11) represents one of the first efforts carried out to jointly analyze several data types, including changes in DNA, RNA expression levels and epigenetics in cancer. Recently, similar multi-omics AML data was made available in combination with drug responses (12). Efforts are ongoing to expand this approach to other hematologic malignancies, many of which are rare on population level. Therefore, integrating different studies for investigating how the identified molecular subtypes compare and relate to survival, or likelihood to respond to different treatments is important.

We propose here a framework aimed to identify and overcome key challenges in analysis of multi-study and multi-platform data and benchmark the methodology with integrative analysis of hematologic malignancies.

## METHODS

### Workflow for integrating different gene expression studies

We exemplify the analysis of data originating from different studies using a dataset of 9,544 gene expression profiles from the Gene Expression Omnibus (GEO) database (1), comprising patient samples representing different cancers and proliferative disorders of hematopoietic lineage origin, cell lines and normal blood cell types. We refer to this sample set as Hemap in the following text. The curated sample annotations and disease categories are available at http://hemap.uta.fi. These data represent microarray data from the commonly used hgu133Plus2 platform that were jointly processed using a typical data analysis pipeline, including the RMA probe summarization algorithm (13) with probe mapping to Entrez Gene IDs (from BrainArray version 18.0.0, ENTREZG) to generate gene expression signal levels and a bias-correction method (5) developed for clinical microarray data to correct for technical differences between studies. To define new molecular subtypes in an unbiased manner, dimensionality reduction was combined with clustering. Initial comparison of the different dimensionality reduction methods encouraged the selection of t-SNE method, specifically the Barnes-Hut approximated version of t-SNE implementation (BH-SNE) (14) to serve as a benchmark scenario for data-driven exploration of disease subtypes. For clustering the data following the dimensionality reduction, we chose the kernel density-based algorithm known as mean-shift clustering (15) (LPCM-package in R). The bandwidth parameter was set to 1.5 (subsets of data, one cancer type) or 2.5 (all data). This method allows the discovery of sample sets which share similar features without having to pre-specify the number of clusters. The term ‘cluster’ is used in the text to refer to this computational clustering result, and the term ‘group’ is used in context of visual examination.

### Pre-processing of additional datasets

From the same microarray platform, 98 biological replicate samples from studies included to Hemap, and 108 samples from an independent study GSE49032 (16) were left out from initial data processing and used as validation data (refer to Supplementary Table S1). These data were normalized as similar as possible to that applied to the original data. Ideally, RMA summarization (13) and bias correction (5) of new samples should be performed together with the original samples. However, considering the dimensions of the original data (9,544 samples), co-normalization of new and old samples is not convenient from the viewpoint of memory usage and computational complexity. In addition, regeneration of the full data matrix would also require re-running all the downstream analysis to maintain the consistency of the results. Rather, we revised the pre-processing approach that allows normalization of new samples to the space of original data. The background correction step of RMA was performed in a standard way as it requires no inter-sample information. The quantile-normalization step, however, utilizes information across all samples. Therefore, we used the normalized distribution from the original data to ensure that data distributions for novel samples do not differ from those of the original ones. In the median polish summarization, the row (probe) effect of the original data was used instead of calculating it across the novel samples. In the bias correction step of novel samples, the coefficients describing the dependency between the bias metrics and gene expressions were obtained from the original data set. It should be noted that all the samples to be normalized should also meet the quality control requirements that were used with the original data set.

A microarray dataset (17) from a different array platform (hgu133a+b) and RNA-seq data from the The Cancer Genome Atlas (TCGA) (11) and BeatAML (12) studies were included to benchmark robustness in discovery of molecular subtypes and addition of samples from different measurement platforms. Data from hgu133a and b platform (17) was normalized using RMA with probe mapping to Entrez gene IDs as above. The TCGA RNA-seq data (11) was obtained through cghub and realigned to hg19 genome using Tophat2 (18) version 2.0.12 with default parameters. The expression of genes included in the microarray was calculated by counting the reads aligning to the corresponding probe regions. RNA-seq data were further processed by log2 transformation. The count matrix for the BeatAML RNA-seq study was obtained from the authors. Genes with expression > 1 cpm in more than 1% of samples were kept and data was normalized using limma voom and quantile normalization.

### Quantitative metrics for assessing dimensionality reduction and clustering results

Existing metrics for evaluating dimensionality reduction results include trustworthiness and continuity (19), analogous to precision and recall in classification. Trustworthiness measures whether data points appearing similar (proximate) in the lower dimensional visualization should be trusted to be similar in actuality, while continuity can be used to assess whether all original proximities ideally would become visualized close-by.

To account for both biological and technical differences that are characteristic of sample sets generated by different studies, we propose new metrics based on normalized mutual information (20) (NMI) to guide the feature selection for dimensionality reduction in context of such heterogeneous data. NMI is defined as MI(X,Y) / ((H(X) + H(Y)) / 2), where MI = mutual information, H = Shannon entropy. X and Y correspond to cluster and class label vectors, respectively. Empirical probability to observe a specific label or cluster was used in calculating MI and entropy.

*pNMI*: This metric was calculated to assess how well the obtained sample clustering (X) can distinguish known biological subtypes (Y, phenotypes) based on NMI between cluster assignment and class labels (maximized).
*eNMI*: Similar as above, NMI was calculated between cluster (X) and experiment (Y, data series) identifiers. The data series differences represent mainly technical (not biological) differences between samples and therefore this measure was minimized.
*cNMI*: In general, we would like to maximize the separation by phenotype (pNMI) and at the same time minimize the separation by data series (eNMI). We combine these two metrics to one by (pNMI + (1 - eNMI)) / 2. Higher values of cNMI indicate better biological separation compared to technical separation and thus this measure was maximized.

The utility of the new metrics was demonstrated by comparing the parameters for t-SNE that uses by default Principal Component Analysis (PCA) (21) for initial reduction of features. We compared this setting to selection of genes based on variance, or their combination. Selecting of 20 to 200 principal components (PCs), 2.5 to 100% of the most variable genes were tested. As another alternative, PCA and clustering based on k-means algorithm were used.

### Remapping of samples to the t-SNE maps

New samples were mapped to an existing t-SNE space by using a modified version of the BH-SNE (14) implementation. The embeddings for existing samples were given as an additional input (the original algorithm would initialize them by sampling from a Gaussian distribution). These embeddings were kept locked throughout the run of the gradient-descent optimizer, while the embeddings for new samples were computed in parallel and independently of each other. In this manner, embeddings of new samples do not affect each other or the embeddings of locked samples, thus preserving the structure of the established t-SNE map. The parameter theta that is related to the distance approximation was set to 0.3 instead of the default 0.5. To remap samples that could come from different measurement platforms, Euclidean distance was replaced by correlation as a distance measure during remapping (one minus Pearson correlation between samples). The t-SNE method minimizes the divergence between two distributions: a distribution that measures pairwise similarities between the original data objects (by default Euclidean distance) and a distribution that measures pairwise similarities between the corresponding points in the embedding. By using a distance metric based on correlation, the similarities between RNA-seq samples and different microarray samples could be estimated without further transformations or normalizations of RNA-seq data.

### Evaluation of the remapping approach

To assign the new samples into existing clusters, distance to the closest centroid was used in cluster assignment. The cluster annotation was determined based on majority voting (for Hemap AML, we utilized here the subset of samples with RNA-seq counterpart that had most uniform annotations across patients). Accuracy was calculated based on the proportion of correct phenotype assignments based on clinical annotation data. In the *in silico* experiments, a subset of samples assigned to specific clusters were removed and then re-mapped. In this case, the original cluster assignments were used as class labels. The 15% most variable genes, t-SNE map and mean-shift clustering were re-calculated for each sample subset, in order to simulate situations when the original sample set represented the molecular subtype (cluster) well, or poorly.

To compare remapping success to a supervised approach, a random forest classifier was trained using 15% most variable genes (randomForest R-package version 4.6–14) by setting the number of trees grown to 20000 while the rest of the parameters were left unchanged. Remapped (removed) samples were used as test data and prediction accuracy was measured. Notice that the labels for training and test data were obtained from clusters found from the full dataset (unlike for re-mapping simulation), and only the 15% most variable genes was re-calculated for each subset to train the classifier.

### Correspondence between t-SNE-map clusters

Similarity in sample clustering between t-SNE maps was evaluated in a data-driven manner using gene set variation analysis (GSVA) (10) enrichment scores. Two gene sets (20 top ranked positively or negatively correlated genes, separately) were defined for each t-SNE map cluster based on significant Pearson correlation (*P*-value < 0.05) of gene expression level and a given cluster label to assess the robustness of the clustering and correspondence between t-SNE maps.

### Gene set analysis

The gene lists for the characterization of sample clusters were obtained from MsigDB v5.0 (9), Wikipathways (06.2015) (22), Recon 1 (23), Pathway Commons 7 (24) and DSigDB v1.0 (25). Gene sets were limited to contain between 5 to 500 expressed genes per gene set, resulting in 19,680 gene sets that were evaluated across the dataset. In addition, gene sets were defined on basis of significant cluster correlation. The gene set variation analysis (GSVA) (10), available in the R/Bioconductor package GSVA 1.13.0, was used to assign a gene set enrichment score (positive for increased and negative for decreased expression) in a sample-wise manner with the following settings: mx.diff = F, tau = 0.25, rnaseq = T if RNA-seq, otherwise rnaseq = F. Empirical *P*-value was computed using 1000 random permutation of genes within the gene set. Estimation of significance was limited to a range of gene set sizes (5–20, 25, 30, 40, 50, 75, 100, 200, 300, 400, 500) to adequately account for differences in gene set size distribution. The observed pathway score was compared with the random permutations of a corresponding gene set size and empirical *P*-value computed as the number of higher/lower scores in the permuted set divided by the total number of permutations. Enrichment of significant scores in a specific cluster was computed using a hypergeometric test. For separate t-SNE maps, consistent enrichment results were obtained by requiring the same directionality in the correlation (to corresponding cluster) with a nominal *P*-value < 0.05 for the smaller data set and adjusted *P*-values < 0.001 for the larger dataset.

### Characterization of sample clusters based on TCGA and BeatAML multi-omics features

Categorical or binary annotation features (including clinical variables), continuous/discrete numeric expression values and continuous/discrete numeric molecular data (mutation, CNV and chromosomal translocation) were collected for each sample. Missing values were marked as NA. Each t-SNE map cluster and combinations of clusters (binary feature) was tested against all other features and the statistical significance evaluated using Spearman correlation between a binary cluster feature and a numeric feature. With binary-binary pairs Fisher's exact test was used. Multiple hypothesis testing correction was performed using the Benjamini-Hochberg (BH) method.

For BeatAML (12) drug analysis, 25 drugs with lower quartile of IC50 value below 10 nm were excluded as these drugs have limited efficacy. Also Flavopiridol (positive control) was excluded, resulting in 96 drugs used in the analysis. One-way Anova was used to find drugs with a differential response between disease subtypes. Multiple hypothesis testing correction was performed using the Bonferroni method.

Myelodysplastic syndrome (MDS) signature was generated using Hemap dataset by identifying genes that were most specific to MDS (hypergeometric test adjusted -log10 *P*-value > 100) resulting in 151 genes (Supplementary Table S4). An additional filtered list was created based on differential expression between prior MDS versus no MDS clinical annotation in BeatAML data (Wilcoxon rank sum test, BH adjusted *P*-value<0.05). Both gene sets were evaluated using GSEA in the TCGA cluster 4 matched AML samples versus remaining AML samples in all three datasets (TCGA, BeatAML and Hemap AML).

### Kaplan-Meier survival analysis

Survival time and status for each TCGA AML sample was obtained from the supplementary table from the original publication (11) (‘Patient Clinical Data’ dated 3.31.12). The R package ‘survival’ was used to compute Univariate Kaplan Meier curves for each TCGA cluster and to calculate the log-rank test.

### Discretizing methylation signal levels with mixture models

Gaussian finite mixture models were fitted by expectation-maximization algorithm provided in the R package mclust (26) (version 4.3) to identify whether the value obtained for a given methylation probe belonged to the signal (expressed) or noise distribution in each sample. The model was chosen by fitting both equal and variable variance models and ultimately choosing the model which achieved a higher Bayesian Information Criterion (BIC) to avoid overfitting. A model with three components was fitted by choosing either an equal or unequal variance model according to BIC. After model fitting, the percentages of measurements belonging to each component were calculated for each sample. The portions of highly methylated regions in the studied cluster were compared against the rest of the samples by calculating the Mann-Whitney U test.

### Code availability

All custom code for analysis are accessible via GitHub https://github.com/systemsgenomics/t-sne.cancermaps. For step-by-step instructions and normalized data matrices (RData files) to reproduce the analysis presented, refer to the User Guide (see Supplementary Material).

## RESULTS

### Framework for multi-study integrative analysis

**Figure 1.**
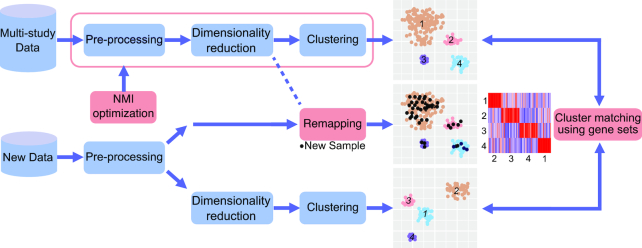
Data integrative analysis workflow. The schematic illustration depicts a typical data-driven analysis workflow and the data integrative approach for multi-study and multi-platform datasets developed here. NMI (Normalized Mutual Information) optimization is used to find an optimal trade-off between biological and technical differences for multi-study data. New data can be added to the same sample stratification based on remapping (applicable to t-SNE maps) or by matching clusters obtained in separate analysis using gene sets.

To develop robust data integrative solutions, we selected a microarray dataset that was collected across several gene expression studies profiling different hematologic malignancies (referred to as Hemap dataset, see **Methods**). The dataset was jointly processed using a typical data analysis pipeline that included established normalization (13) and batch effect correction (5) steps in data pre-processing, followed by dimensionality reduction using the t-SNE method that has been successfully applied in context of both simulated and real datasets (14,27), and finally a clustering step (15,28) to define disease subtypes in a data-driven manner. Our framework, depicted in Figure 1, includes new quantitative metrics to guide the choice of analysis parameters to minimize batch effects that arise from different laboratory protocols, allows incorporation of additional samples to lower dimensional representations obtained with t-SNE (27) and utilizes gene-set based integration between multi-omics studies and platforms.

### Assessing the contribution of technical variation in sample separation

**Figure 2.**
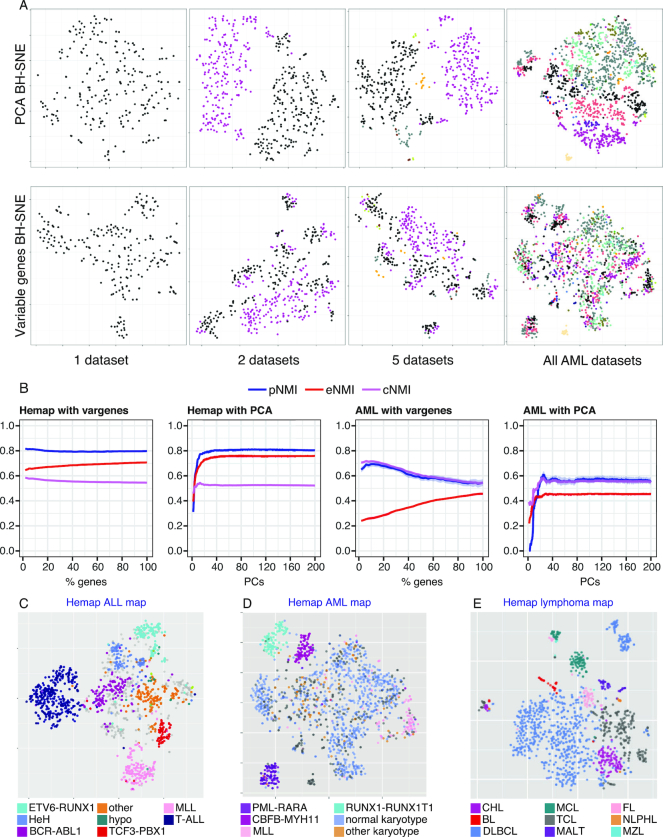
Assessing the contribution of technical variation in sample clustering. (**A**) Iterative addition of AML data series to the sample set used for t-SNE is shown. Each dot represents one sample and colors correspond to study that generated the data. The difference between using the PCA pre-processing step (above) or using 15% of most variable genes (below) is visualized from a succession of maps (a single data series, 2, 5 or all). (**B**) The metrics for separation of phenotypes (pNMI, in blue), data series (eNMI, in red) and combined metric (cNMI, in violet) shown represent the mean of 100 permutations with different seed selections. Standard deviation is shown in grey around the lines. Selecting a different number of principal components (PCs) is compared to selecting a different percentage of variable genes using the full Hemap dataset and Hemap AML sample set. The fine structure on the t-SNE maps with 15% gene selection matches closely pre-B-ALL (**C**), AML (**D**) and BCL (**E**) clinical subtypes (in color).

A fundamental challenge for joint analysis of the genome-wide data available in public repositories is the technical variability between data generated by different laboratories. This challenge is exemplified by the application of t-SNE across the heterogeneous data, with the subset of Hemap AML samples: Upon iterative addition of experiments, as shown in Figure 2A, analysis using the default PCA pre-processing step results in separation of the data based on the study rather than biological class (Figure 2A, PCA BH-SNE). Testing alternative solutions (see **Methods**), we found that selection of genes based on variable expression results in a much better sample grouping that does not reflect data origin (Figure 2A, Variable genes BH-SNE). The technical bias in the PCA BH-SNE result, was not apparent based on two common metrics, continuity and trustworthiness (19) (PCA BH-SNE 0.92 and 0.96; Variable genes BH-SNE 0.95, 0.97, respectively). To address this issue, we defined new quality measures for quantitative evaluation of the sample clustering in context of heterogeneous datasets: pNMI that captures the separation of phenotypes (maximized), and eNMI that can be used to penalize the separation of data by experiment (minimized) (see Methods). The calculation of these metrics (or the combined metric, cNMI) requires that at least some samples have an annotated class and that origin (study/experiment identifier) of the samples is known. Using these metrics, the effect of different analysis choices, such as choosing different number of principal components, or different percentage of genes could be systematically assessed for the AML subset and the full sample collection, as illustrated in Figure 2B (see also Supplementary Figure S1). In both cases, feature selection based on genes with highest variance performed favorably, reducing the technical biases (notice that the pNMI values between AML and all Hemap samples are not directly comparable since clinical subtype information was used for AML, whereas for all samples pNMI was defined based on the main disease type). This motivated the selection of 15% most variable genes for t-SNE analysis in this study. However, the same approach could also be used to test whether the observed technical effects could be diminished for example by additional or alternative pre-processing steps (Figure 1). The annotated sample category is visualized in Figure 2C–E on t-SNE maps generated based on most variable genes (see also Supplementary Table S2), including two other sample subsets (acute lymphoblastic leukemia (ALL) and lymphomas). In each map, a sample grouping driven by cancer subtypes was obtained. Therefore, the quantitative assessment based on the new metrics can guide parameter selection for unsupervised sample stratification methods to mitigate technical variation effects.

### Incorporation of additional datasets to predetermined t-SNE space

**Figure 3.**
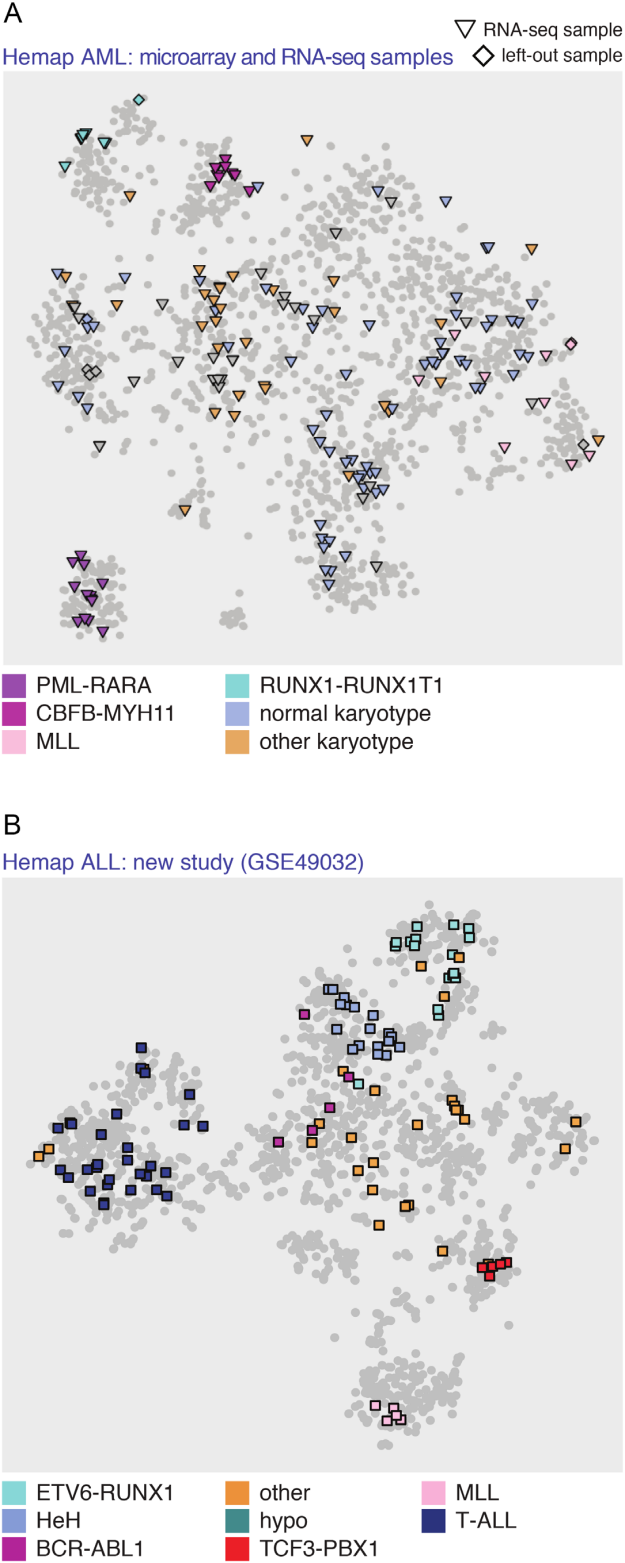
Remapping new samples to the t-SNE map. (**A**) AML microarray validation samples that were left out (*N* = 10, diamonds) and TCGA RNA-seq samples (*N* = 162, triangles) re-mapped on the Hemap AML t-SNE map. Notice that similar samples mapped in close proximity to each other may overlap on the visualization. (**B**) Remapping result for samples from an independent ALL study^11^ (GSE49032) to the Hemap ALL t-SNE map. The subtype of re-mapped samples is indicated in color in **A** and **B**.

Next, we developed a remapping algorithm that allows additional samples to be included to the existing map. In this manner, the obtained clustering of disease (sub-)types on the t-SNE maps could be used to characterize new samples. Since many new studies currently use RNA-seq, the remapping algorithm was extended to incorporate alternative types of data by revising the similarity metric (see Methods). As a first benchmark, the left-out samples (*N* = 10 from AML, and in total *N* = 98 Hemap samples) were all assigned to the same cluster as their replicates, as shown on the Hemap AML t-SNE map in Figure 3A in diamond shapes (see also Supplementary Table S3 and Figure S2 that shows the successful association of all 98 validation samples with the disease-of-origin). Secondly, re-mapping an independent microarray dataset to the ALL t-SNE-map successfully assigned the clinical subtype for 95% of samples (Figure 3B). Next, RNA-seq samples were analyzed from the TCGA AML study. For 79 cases (from 162), a corresponding microarray sample exists in the Hemap AML sample set. Remapping success was first evaluated by comparing the cluster assignments between the microarray and RNA-seq sample pairs: 81% were assigned to the same cluster center. We then considered the remaining RNA-seq samples. Of these patient RNA profiles, 97% were placed to a cluster matching the annotated category. Remapping success for all TCGA AML samples is shown in Figure 3A and Supplementary Table S3.

For remapping to succeed, the initial dataset should be representative of the biological heterogeneity in the disease. Therefore, as a final benchmark we evaluated how robust our method was in situations where a subtype was poorly represented in the initial dataset. To simulate this scenario, samples from a specific cluster were iteratively removed. We compared the well-separated AML cluster 14 (with PML-RARA samples) and cluster 4 (including CEBPA-mutated samples) that was more challenging to distinguish (see Supplementary Figure S3 and Table S2). The re-mapping of samples from cluster 14 achieved similar accuracy as supervised random forest classifier (see **Methods**), even when only 10 samples were included in the initial data (accuracy 0.87), while the accuracy for cluster 4 varied from 0.93 to 0.66. In this case the supervised classifier trained with the original clusters was more robust, however it should be noted that the clusters were re-calculated from each subset only for the re-mapping (Supplementary Figure S3). Finally, to exemplify whether it would be possible to distinguish that the subtype for the remapped sample is missing in the initial dataset, we included diagnostic plots on the correlation scores across samples re-mapped with cluster-specific high, median, and low correlation, and for the situation that the cluster was completely missing (Supplementary Figure S4).

In conclusion, our re-mapping method readily extends t-SNE results beyond the initial sample set when new samples matching the represented biological subtypes are analyzed, and shows robustness for different technologies and matching underrepresented subtypes.

### Comparison of data-driven stratifications of molecular disease subtypes

**Figure 4.**
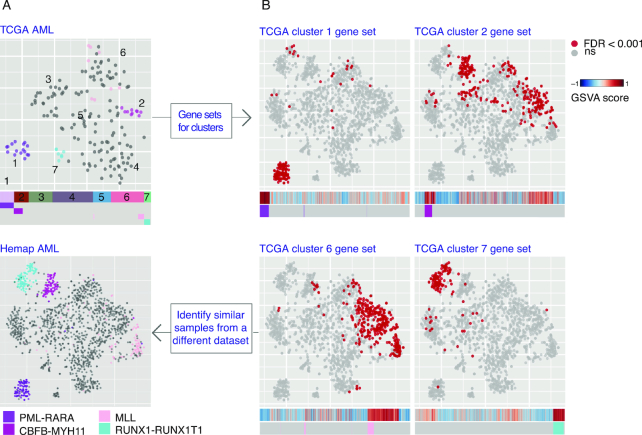
Evaluation of disease subtypes across datasets. Comparison of sample clustering on the t-SNE maps for TCGA AML RNA-seq samples (*N* = 162) and Hemap AML is shown. (**A**) The data-driven cluster assignment (TCGA clusters 1–7) can be compared with sample molecular annotations colored on the map and the heatmaps below. (**B**) Enrichment scores for TCGA cluster gene sets that matched samples with common fusion genes (clusters 1, 2, 6 and 7) are colored on the Hemap AML map (significant enrichment adj. *P*-value < 0.001 in red). The raw GSVA scores are shown below as a heat map (red tones indicate high expression of the gene set and blue tones low expression).

Comparisons between studies would allow to determine whether the same molecular subtypes segregate in a reproducible manner. Towards this end, we developed methodology for examining the correspondence between clusters on separate t-SNE maps. We chose the TCGA AML (11) (RNA-seq, Figure 4), Ross ALL (17) (microarray, Supplementary Figure S5) and BeatAML (12) (RNA-seq, Supplementary Figure S6) studies for comparative analysis. Figure 4A shows the generated t-SNE map for TCGA RNA-seq samples, which resulted in seven distinct clusters (referred to as TCGA clusters 1–7). The comparison with annotated cytogenetic types was consistent; samples carrying PML-RARA, RUNX1-RUNX1T1, CBFB-MYH or MLL fusions segregated into distinct clusters. To identify matching clusters from the Hemap AML t-SNE map, we developed a data-driven approach that defines and scores gene sets for each cluster (see **Methods**, refer to Supplementary Table S4 for TCGA cluster gene sets). The samples with significant enrichment are colored on the Hemap AML t-SNE map, and their enrichment score compared to annotations as a heatmap (Figure 4B). Samples with matching fusion gene status received the highest enrichment scores, allowing matching of clusters between the maps (Supplementary Table S5). The application to ALL (17) and BeatAML (12) samples (Supplementary Figures S5 and S6, respectively) showed similar robustness in discovery of matching subtypes.

### Integration with multi-omics profiles and pathway activity analysis to characterize the discovered molecular subtypes

**Figure 5.**
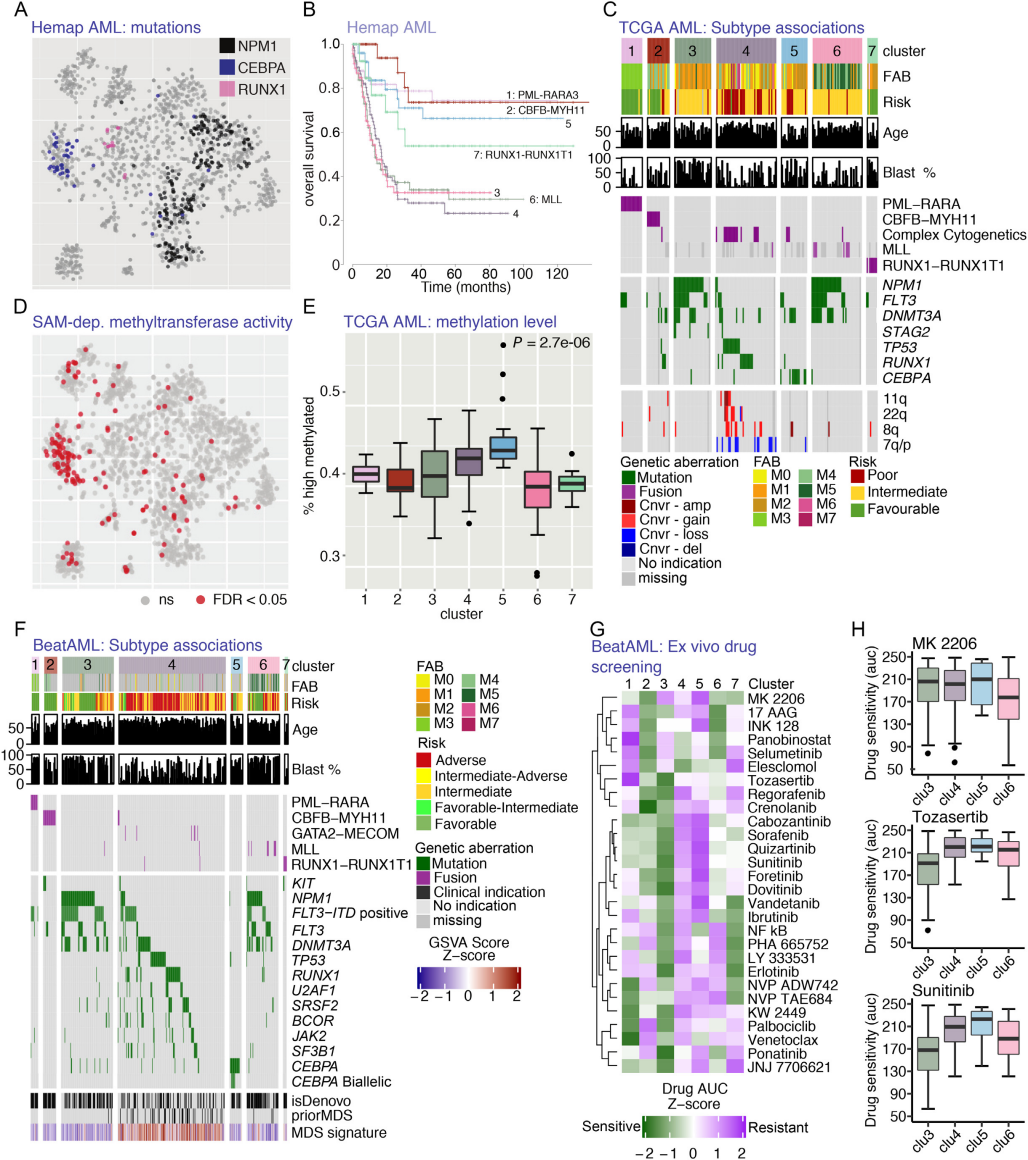
Multilevel data integration reveals AML molecular subtypes with distinct mutation and epigenetic phenotypes. (**A**) Mutational status for most significantly cluster-associated mutations is indicated in color on the Hemap AML map. (**B**) The survival analysis of Hemap patients comparing the subtypes matched with the TCGA map are shown as Kaplan-Meier plots. (**C**) Heatmap of significant genetic and clinical associations (Fisher′s exact test or correlation test of significance adj. *P*-value<0.05) for each subtype is shown. (**D**) Significant enrichment (adj. *P*-value < 0.05) for SAM-dependent methyltransferase activity is shown on the Hemap AML t-SNE map. (**E**) The percentage of regions assigned to the high methylation state is visualized as box plots comparing TCGA cluster 5 patients (*N* = 20) and rest of TCGA AML clusters (Mann-Whitney U-test *P*-value is indicated). (**F**) Significant associations (Fisher′s exact test or correlation test of significance adj. P-value<0.01) for BeatAML samples matching TCGA clusters are shown as in **C**. (**G**) The median drug AUC values per cluster from BeatAML ex-vivo drug screening are shown as a heatmap for drugs with differential sensitivity across AML molecular subtypes (One-way Anova, adj. *P*-value<0.05). **H**. Drug AUC values for MK 2206 (Akt inhibitor), Tozasertib (Aurora kinase inhibitor) and Sunitinib (FLT3 inhibitor) are shown as boxplot for normal karyotype clusters 3,4,5 and cluster 6 also containing FLT-mutation positive samples.

The classification of AML has traditionally distinguished between fusion gene-positive categories (29,30). However, additional clusters segregate in the TCGA and Hemap AML t-SNE maps that can be matched between the studies (Supplementary Figures S7A–C, Table S5). *NPM1, CEBPA* and *RUNX1* mutations were characteristic for these non-fusion samples in Hemap (Figure 5A). The distinction between the samples is clinically relevant, as the identified clusters differed in overall survival (Figure 5B). Statistically significant associations between the map clusters and different multi-omics features or pathway activity scores were queried to further characterize the discovered molecular subtypes (see **Methods**). Based on correlation of cluster assignment with multi-omics TCGA data (11), *NPM1* mutations were associated with two clusters (TCGA clusters 3 and 6), whereas *CEBPA* mutations characterized TCGA cluster 5 (Figure 5C). The distinction between these two NPM1 positive clusters was associated with the cellular morphology (FAB type in Figure 5C). In addition, our analysis revealed a subgroup (TCGA cluster 4 and corresponding Hemap samples indicated in Supplementary Figure S7D) with several cases positive for either *TP53* or *RUNX1* mutations and/or complex karyotypes (Figure 5A and C).

Next, we detected significant associations between the cluster category and pathway activity. The gene set enrichment analysis (see **Methods**) confirmed that the TCGA and Hemap patients from the matched clusters share molecular characteristics (Supplementary Table S5). The TCGA cluster 5 (*CEBPA* mutated, Figure 5C) and its corresponding cluster on the Hemap AML map (refer to Figure 5A and Supplementary Figure S4B) were significantly associated with S-adenosylmethionine (SAM)-dependent methyltransferase activity (Figure 5D, hypergeometric test adjusted *P*-values 5.6e-05 and 1.5e-26, respectively). Because all DNA methyltransferases use SAM, we quantified TCGA DNA methylation data to test whether global changes in methylation patterns exist between clusters (see **Methods**). Accordingly, we observed a significant elevation in DNA methylation level in the TCGA cluster 5 compared to other samples (Figure 5E, Mann-Whitney U-test *P* = 2.681e-06).

Corresponding genetic and clinical features characterizing the AML molecular subtypes could be robustly identified also from BeatAML data (Figure 5F). In comparison, mutations in *IDH1/2, TET2, NRAS* and *KRAS* were significantly enriched in several subtypes, but not subtype specific (Supplementary Figure S7E). TCGA cluster 4 matched in BeatAML data a cluster where several cases had prior MDS clinical status and a secondary disease. Accordingly, an MDS gene expression signature (built using MDS samples in Hemap) was enriched in samples associated with this cluster in all data sets (Figure 5F and Supplementary Figure S7F–H).

Encouraged by the robustness of our subtype characteristics across data sets, we examined the drug response AUC values available for the BeatAML samples. In total 28 drugs were differentially sensitive (one-way Anova test, adjusted *P*-value <0.05) (Figure 5G) comparing the discovered AML subtypes. Samples matched to TCGA cluster 3, with high blast count (Figure 5C and F), were sensitive to most drugs. However, samples matched to each of the non-fusion TCGA clusters (3, 4 and 5) were resistant to MK 2206 (AKT inhibitor) (Figure 5H). In comparison TCGA cluster 6 that resembled cluster 3 based on mutations (Figure 5C and F) but had a more differentiated cell phenotype was associated with cases that were sensitive (Figure 5H), further supported by various AKT pathway signatures that were enriched in this cluster (Supplementary Table S5). The Aurora kinase inhibitor Tozasertib and the FLT3 inhibitor Sunitinib were effective in cases matched to TCGA cluster 3, while cases matching clusters 4 and 5 were resistant to all FLT inhibitors tested (Figure 5H).

## DISCUSSION

A large number of biological conditions have been characterized at genome-wide level since the introduction of microarray and deep sequencing technology (1–3), However, most of the studies include only tens to hundreds of samples. Cancers of hematopoietic origin serve as an important example where data integration is essential from the sample availability perspective, since many of these cancers are rare on the population level (30,31). Therefore, understanding the complete heterogeneity and similarity of disease states and their subtypes requires integrative data analysis methodology. Here, we tested solutions that allow distinguishing sample separation driven by technical variation, evaluating the robustness of the obtained biological stratifications between studies and inclusion of new sample sets and data types to pre-existing analysis results. We demonstrated the relevance of our approach to generate insight into disease biology and to identify potential novel molecular disease subtypes by performing an integrative analysis across three large AML studies.

Currently, it is relatively easy to compile multi-study datasets by prioritizing relevant data sets based on gene-level queries (32). However, removing technical variation, or batch effects, from data before downstream analysis is a vital part of studies analyzing such datasets generated from different experiments (4,5,33). Failure to identify and remove unwanted technical variation may lead to an increased amount of false positive findings (34). We propose here new NMI metrics to guide the choice among alternative methods and settings to achieve minimal loss of biologically relevant gene information (high separation of phenotypes) while reducing the technical variation enough not to interfere with the biological interpretation of the results. While the value range of the NMI metrics is different for each dataset, observing eNMI values above pNMI can serve as a general indicator that technical differences may overwhelm the biological differences in the sample stratification obtained. The NMI metrics extend to more than just microarray and RNA-seq data presented here, to scenarios where sample clustering is relevant. For example, in context of single-cell RNA-seq the challenge of batch effects has been recognized (35), and new data sets are becoming increasingly available.

Motivated by recent advances in unsupervised applications, we selected the t-SNE algorithm for further development, with the aim to provide solutions suitable for heterogeneous data. Evaluating the dimensionality reduction alone (continuity, trustworthiness (19)) was found insufficient to capture the technical bias in the t-SNE maps. Based on the NMI metrics, we found that removing less variable genes prior to t-SNE or clustering could remedy the technical biases. Alternatively, additional pre-processing steps or decomposing the variation could be attempted to reduce batch effects, including methods that attempt to remove the variation contained in top principal components (36,37). To date, microarray repositories are still by far the largest resource for molecular data and hold a vast potential for large-scale studies. Compared to existing transcriptome collections (38,39), our goal was to develop methods that can generalize the integrative analysis beyond the initial dataset. We showed that new samples can be added to extend an existing t-SNE map. This could be relevant for achieving compatibility of analysis against different reference dataset versions. Moreover, we demonstrated that inclusion of RNA-seq profiles for joint analysis with microarray results was feasible in this manner. Secondly, we introduce an alternative gene set-based integration across different AML datasets, using the unsupervised t-SNE projections of different studies as the initial starting point. For remapping or defining cluster gene sets, the robustness of the analysis is related to how representative the initial dataset is of the biological context, such as the cancer subtypes considered here. The overall correlation to samples on the map and cluster-specificity of high correlation values can be used as indication of how reliably the sample can be re-mapped. The enrichment results with GSVA are related to the sample composition, as genes are ranked based on kernel estimation of the cumulative density function using all samples (10). Therefore, the re-mapping approach would be better suited when comparing samples from small or subtype-focused studies to a larger reference study.

Based on these data integrative solutions, we performed a comprehensive analysis with integration of additional data types from two multi-omics AML studies to further characterize the identified sample groups. Our approach considers gene expression levels as the main phenotype that gene set and pathway analysis can further characterize. Other data types such as mutations and methylation levels were included by correlating them with the clusters observed in this phenotype (gene expression) space. Alternatively, multi-view methods designed to preserve structures between several layers of data could be utilized (40,41). While these methods appear promising, in practice different data types and result interpretation present challenges (41). Factor analysis could represent an approach that generates better interpretable data representations when sufficient number of samples is available (42).

Our analysis comparing data-driven identification of AML molecular subtypes from TCGA, Hemap and BeatAML datasets revealed a distinct clustering of *CEBPA, NPM1, RUNX1* and *TP53* mutation positive samples. These molecular phenotypes that we found to distinguish clusters of fusion gene-negative samples could be identified from each dataset, demonstrating that the molecular subtypes found are robust, comparable to the fusion gene clusters distinguished in previous classifications (29,30). The cluster with the worst survival in our analysis included samples that were characterized by complex karyotypes and *TP53* mutations, agreeing with recent genotyping data of recurrent mutations (43). The enrichment of MDS-linked mutations (*TP53, RUNX1, ASXL1*, and *U2AF1*), clinical annotations of prior MDS status and the MDS-like gene expression signature indicate that MDS-like AML cases have a unique molecular phenotype associated with poor survival. Furthermore, the analysis of cluster-associated mutations distinguished a patient cluster with *CEBPA* mutations. Using pathway analysis, we could demonstrate that TCGA and Hemap patients from the matched clusters had elevated expression of genes involved in SAM-dependent methylation activity. Previous studies have found contradicting results, reporting both specific (11) or broad (44) methylation changes. We validated that patients with high pathway activity score had an elevated global DNA methylation level using multi-omics TCGA data. Finally, we linked drug sensitivities to this AML molecular subtype characterization. In this manner, the integrative data analysis presented could facilitate further characterization of drug resistance mechanisms.

In conclusion, we present new data integration approaches for multi-study and multi-omics datasets that allow researching disease subgroups across studies. In future, this analysis framework can be adopted to support the utilization of genome-wide data across different biological systems and disease contexts.

## Supplementary Material

gkz281_Supplemental_Files
