# Supplementary material for: Data-driven characterization of molecular phenotypes across heterogeneous sample collections
Source: Nucleic Acids Res. 2019 Apr 24;47(13):e76. doi: 10.1093/nar/gkz281 (PMC6648337; doi:10.1093/nar/gkz281)
Supplement: gkz281_Supplemental_Files [file gkz281_supplemental_files.zip › supplTextFigs_revised.pdf]

Supplementary material for:

Data-driven characterization of molecular phenotypes across heterogeneous sample collections

Mehtonen J.[1]+, Pölönen P.[1]+, Häyrynen S[2]., Dufva O.[3], Lin J.[2], Liuksiala T.[2,4], Granberg K.[2], Lohi O.[4], Hautamäki V.[5], Nykter M.[2]*, and Heinäniemi M.[1]*

[1]Institute of Biomedicine, School of Medicine, University of Eastern Finland, Kuopio, Finland

[2]Faculty of Medicine and Health Technology, Tampere University, Tampere, Finland

[3]Hematology Research Unit Helsinki, University of Helsinki and Department of Hematology, Helsinki University Hospital Comprehensive Cancer Center, Helsinki, Finland

[4]Tampere Center for Child Health Research, Tampere University and Tampere University Hospital, Tampere, Finland

[5]School of Computing, University of Eastern Finland, Joensuu, Finland

Contents

Supplementary Figures

Supplementary Table legends

User guide for available code and data

Supplementary Figures

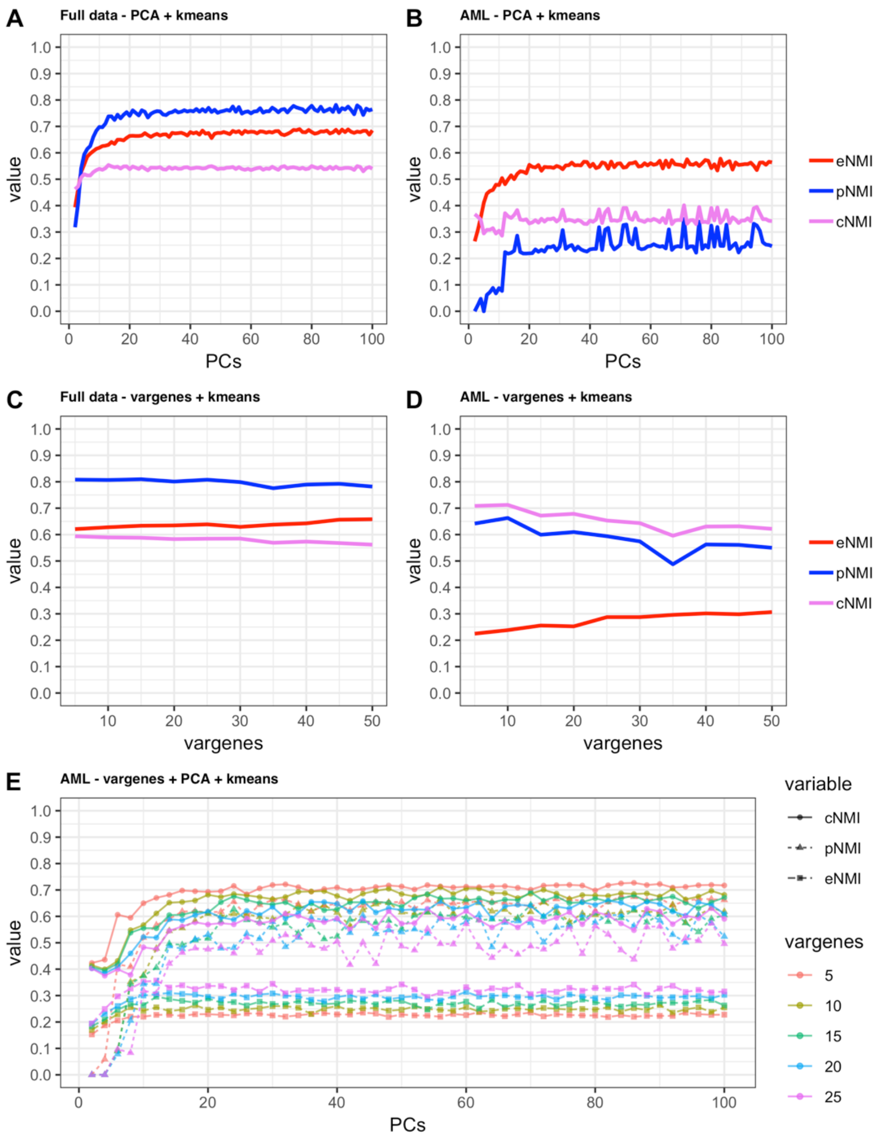

Fig. S1. **Comparison of alternative method or parameter choices for dimensionality reduction and clustersing based on NMI metrics**. Separation based on data series (eNMI) and biological sample class (pNMI), or their combined metric cNMI are compared for the combination of PCA and k-means clustering (**A** and **B**) varying the number of principal components, or performing the clustering (with k-means) directly in the space defined by most variable genes (**C** and **D**). The combination of most variable genes and top principal components is shown in **E**.

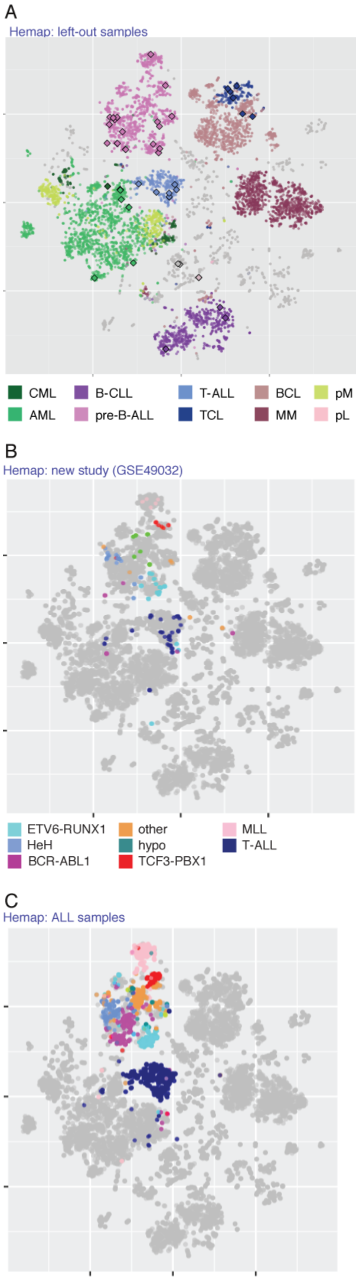

Fig. S2. Remapping in context of all Hemap samples. A. The placement of replicate samples (Left-out sample set, N = 98) is visualized as diamonds on the Hemap t-SNE map. Notice that similar samples mapped in close proximity to each other, leading to overlapping on the visualization. The disease type of re-mapped and Hemap samples is indicated in color (CML, chronic myeloid leukemia; AML, acute myeloid leukemia; B-CLL, B-chronic lymphocytic leukemia; pre-B-ALL, precursor-B-acute lymphoblastic leukemia; T-ALL, T-acute lymphoblastic leukemia; TCL, T-cell lymphoma; BCL, B-cell lymphoma; MM, multiple myeloma; pM, myeloproliferative disease; pL, lymphoproliferative disease. **B**. Remapping result for samples from an ALL study (GSE49032, not included to Hemap). The subtype of re-mapped samples is indicated in color. **C**. The subtype of Hemap ALL samples is indicated in color.

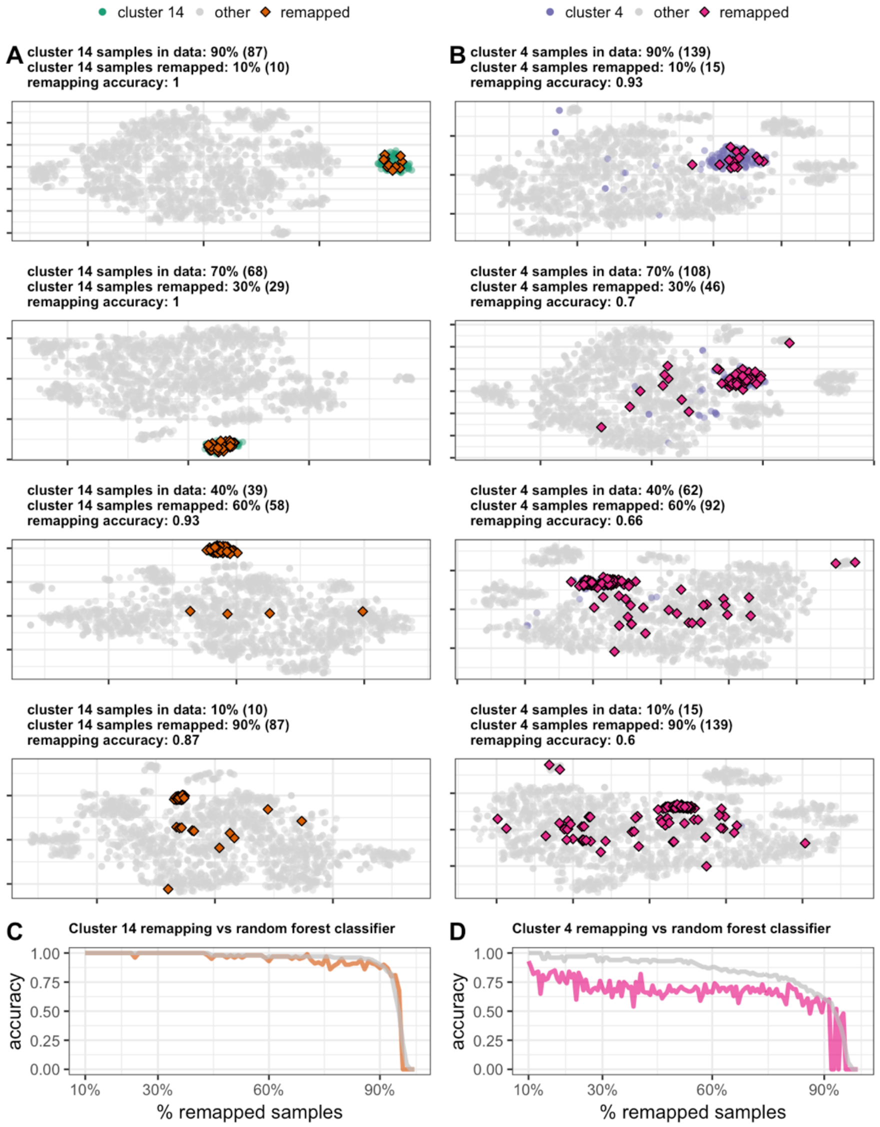

Fig. S3. Evaluation of re-mapping success upon cluster sub-sampling. Samples from clusters 4 (CEBPA-mutated samples, in **A**) and 14 (PML-RARA samples, in **B**) were sub-sampled to 90, 60, 30, and 10% of original samples left in the data and the success in remapping the remaining samples is shown on the t-SNE maps. Notice that the gene subset (15% most variable), t-SNE map and clustering were also re-calculated. **C-D.** Accuracy of the remapping approach (in color) is plotted across different sub-samplings and compared to the accuracy obtained using a random forest classifier (in grey). Only the gene subset was re-calculated for the classifier training.

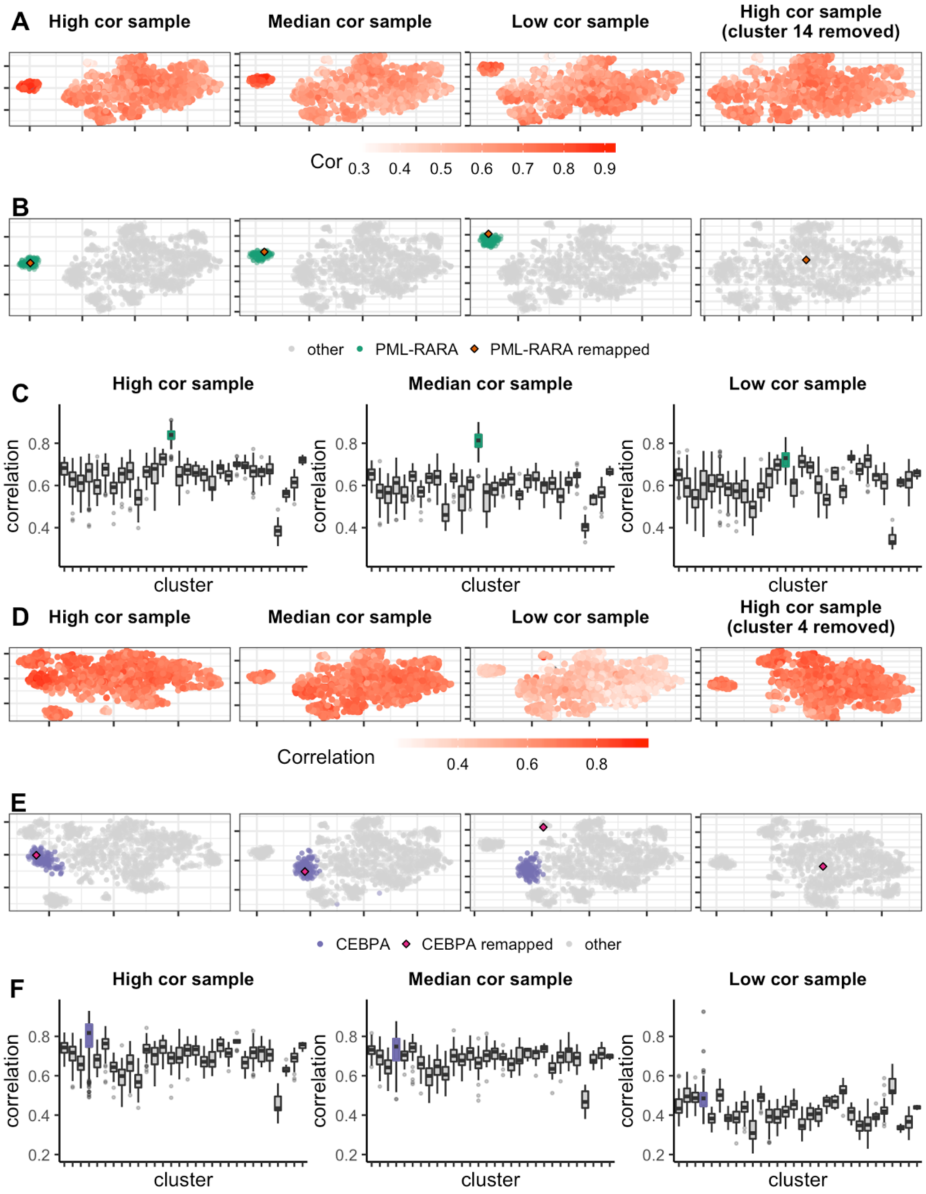

Fig. S4. Evaluating remapping performance based on sample correlations. A. The correlation to samples on the map is shown in color on the t-SNE maps for three re-mapped samples that had high (0.84), median (0.81), and low (0.73) correlation to cluster 14 (PML-RARA samples). Their remapping result is shown in **B**, and the cluster-wise correlation summarized as box plots in **C**. **D-E.** Samples with highest (0.79), median (0.73), and low (0.49) correlation to cluster 4 (CEBPA-mutated samples) are shown as in **A**-**C**. Notice that t-SNE maps were re-calculated with one sample or cluster left out.

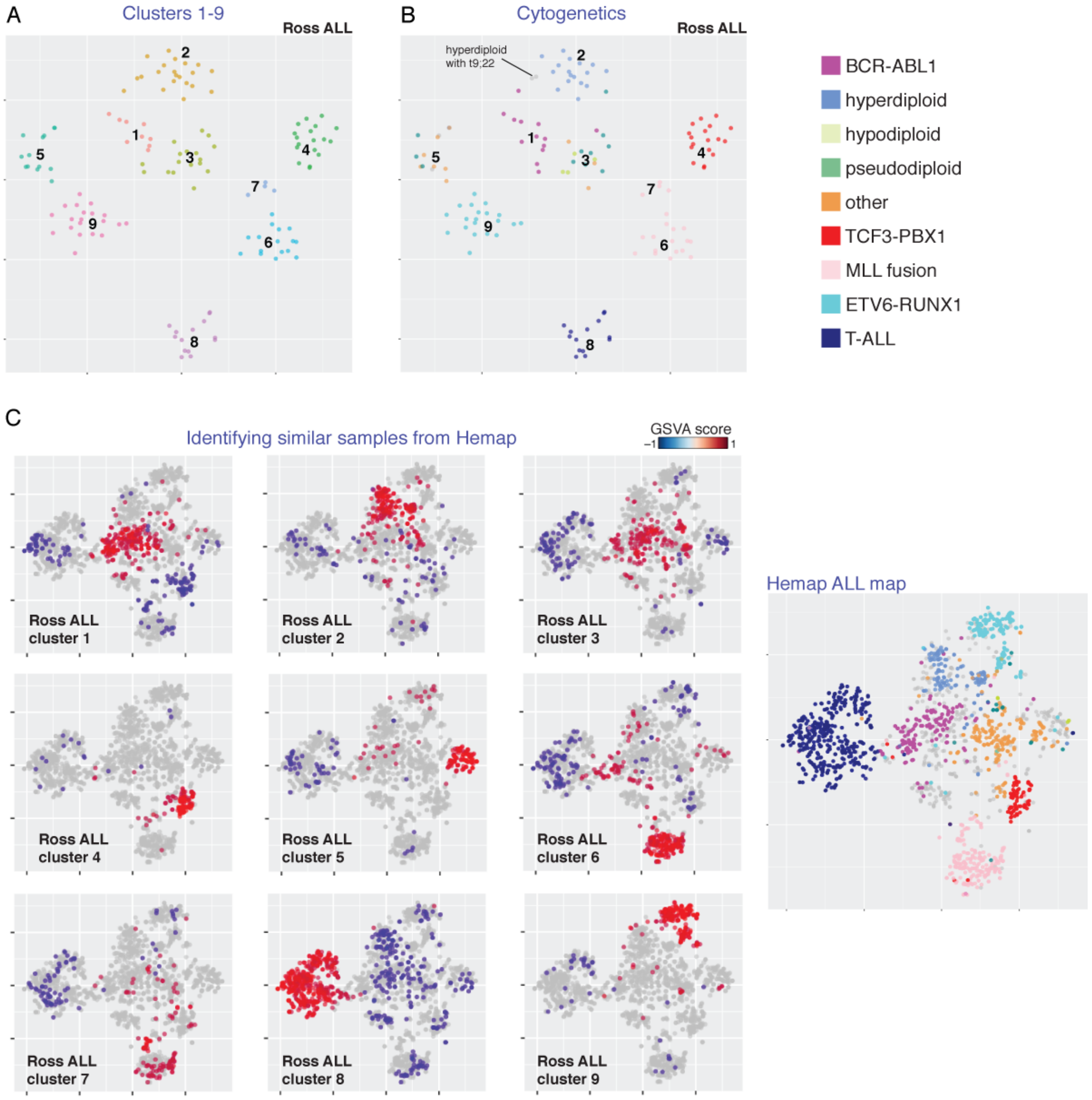

Fig. S5. Comparing an independent ALL dataset with Hemap samples using t-SNE maps. Comparison of samples from an independent ALL study (17) with Hemap ALL samples is shown. **A**. The data-driven cluster assignment (left, colors indicate different clusters) can be compared to annotated cytogenetic types (indicated in color in **B**) and correlated with sample molecular features. **C**. The cluster-specific gene set scores are visualized on the Hemap ALL t-SNE map. The annotated cytogenetic sample type is colored on the map (right) as in **B** for evaluating the correspondence of gene set score based cluster matching.

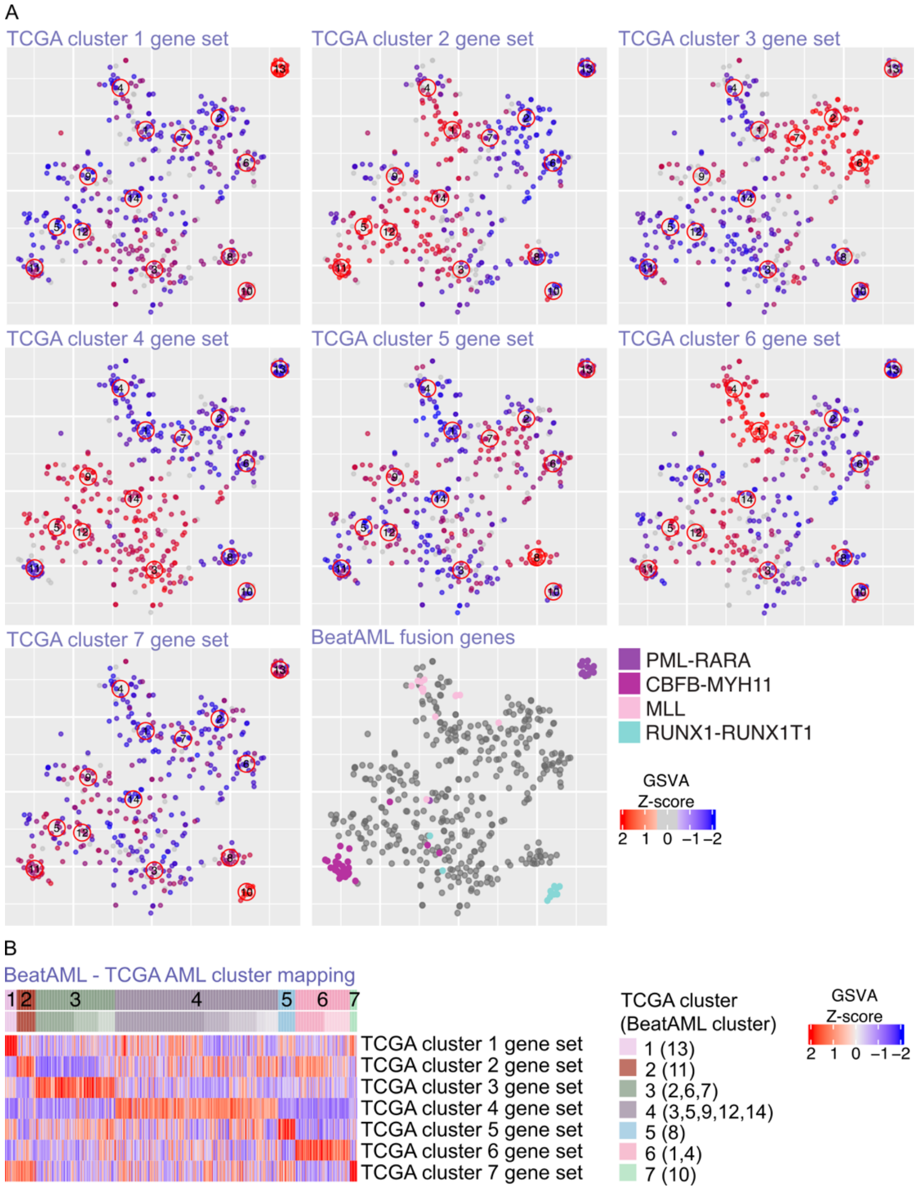

Fig. S6. Matching the TCGA clusters with the BeatAML t-SNE map. **A**. The cluster-specific gene set score for each TCGA cluster (1-7) is visualized on the BeatAML t-SNE map. **B**. A heatmap visualization of GSVA scores for TCGA clusters is shown for the BeatAML dataset. Lighter color shades in the cluster panel above the heatmap indicate the samples from matched BeatAML clusters (listed in brackets on the right).

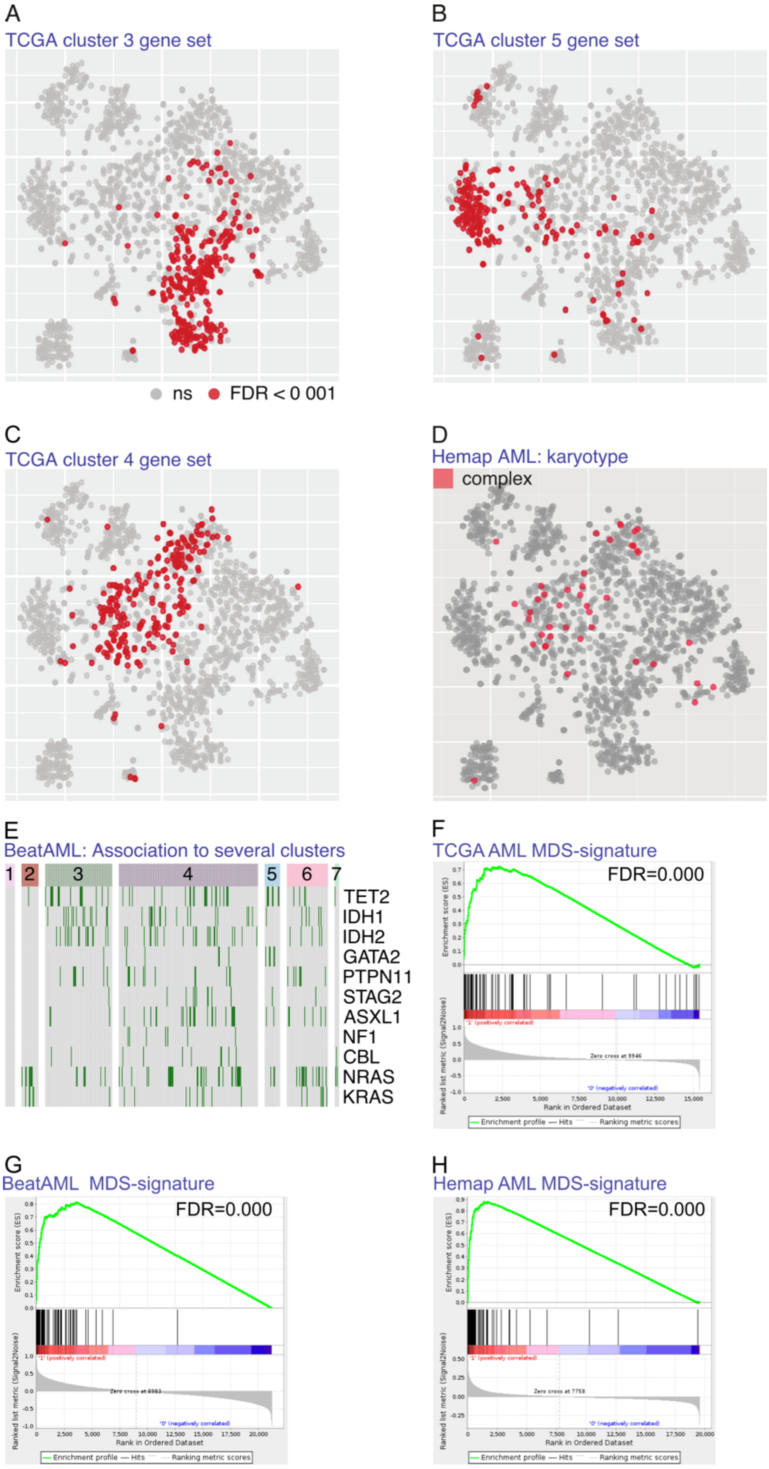

Fig. S7. TCGA AML subtypes 3-5 sample characteristics. **A-C.** Significant enrichment (adj. P-value < 0.001) of TCGA clusters 3-5 gene sets is colored on the Hemap AML map. **D.** Location of complex karyotype samples indicated in color on the Hemap AML t-SNE map. **E.** Additional mutations associated with TCGA-clusters based on BeatAML data integration. **F-H.** The running sum plot of MDS-signature (filtered geneset) enrichment in TCGA cluster 4 matching samples is shown in the indicated AML dataset. Significant enrichment, FDR=0.000, was obtained also with the unfiltered gene set (refer to **Table S4**).

Supplementary Table legends

Table S1. Left-out sample set. Annotations related to disease type and cytogenetics for the left-out sample set.

Table S2. Hemap AML, ALL and lymphoma t-SNE coordinates and sample classification. The Hemap AML, ALL and lymphoma samples were separately analysed, generating t-SNE map coordinates and clustering results for each disease type. Results are presented with per sample annotations.

Table S3. Re-mapped samples. Remapping of left-out samples to the t-SNE map with all samples is presented in the first datasheet. The remaining data sheets describes the remapping to Hemap AML t-SNE map, separately for remapped RNA-seq samples from TCGA with a microarray replicate (datasheet 2), or the remapped RNA-seq samples from TCGA without a replicate (datasheet 3). This distinction was made since a subset of the samples were profiled using microarrays (included to Hemap) and RNA-seq (indicated by the matching between TCGA and GSM sample identifiers in columns 1-2). A cluster was assigned a cytogenetic type based on a majority vote among the annotated classes of RNA-seq samples with microarray counterparts.

Table S4. Cluster-specific gene sets and MDS signature. Gene sets defined in this study are listed. Up to twenty most significantly correlating genes for each TCGA AML t-SNE map cluster (1-7), or Ross ALL study clusters 1-9 used for cluster-matching are shown. An MDS-signatures derived from differentially expressed genes between Hemap MDS samples vs. rest of the samples, or additional filtering with BeatAML differential expression in prior MDS samples, are shown.

Table S5. Molecular characterisation of AML clusters using Hemap, TCGA and BeatAML data. Result tables in datasheets 2-4 have 13 columns: Gene set name (1), Hemap AML cluster (2), Spearman correlation coefficient between gene set and cluster (3), number of samples (4), -log10 correlation *P*-value (5), BH correction factor (6), -log10 correlation adjusted *P*-value (7), cluster enrichment test *P*-value (8), cluster enrichment test adjusted *P*-value (9), Nominal and adjusted *P*-values for correlation and enrichment test in the TCGA AML dataset (10-13). Result tables in data sheets 5-6 have six columns: cluster or cluster combinations (1), clinical associations (2), correlation coefficient (3), P-values from Spearman correlation test for binary-numeric pairs and two-sided Fisher's exact test for binary-binary pairs (4-6). P-values were adjusted using BH method. Data sheet 6 contains Anova results from comparison of 97 drug *ex-vivo* responses to identify differential drug responses between samples matched to TCGA clusters 1-7 in BeatAML data. P-values were adjusted using the Bonferroni method. TCGA clusters matched with Hemap AML map clusters are listed in the last data sheet. Spearman correlation coefficient and adjusted P-value are shown for up- and downregulated gene sets.

User guide: Characterization of molecular phenotypes across heterogeneous sample collections

Background

The methods presented in this user guide were developed for multi-center and multi-platform datasets to allow distinguishing technical variation, evaluating the robustness of the obtained biological stratifications via comparisons between studies and inclusion of new sample sets and data types, as they become available. Benchmarking data from hematological malignancies (Hemap dataset) and source code is available for download, together with installation instructions for required software. The required objects for three example use cases are available as RData files. The dimensionality reduction method t-SNE is used here to obtain data-driven sample stratifications, however Use case 1 and 3 can easily adopted to use alternative methods for sample clustering.

Script availability

https://github.com/systemsgenomics/t-sne.cancermaps

Data availability

https://drive.google.com/open?id=18i6EPHNDJyrAfZTLadKWfHQt3NxNd_za

t-SNE map generation, evaluation and sample remapping

Description of each parameter can be found at the end of the document.

1) To generate a t-SNE map from the required objects, use the provided function CancerMap. For parameters in capital letters, refer to the end of the document. Output is a dataframe (*X*) with columns id, x, y and cluster.

```
CancerMap(t(matrix), NAME, VAR, BW, PATH_OUTPUT)
```

2) Evaluation of method and parameter choices. At this step it is wise to verify that the separation of the samples (e.g. clusters in a t-SNE map) reflects biology and not the data provider. We recommend to use established molecular subtypes for the disease in question. See use case 1 for detailed example analysis.

Calculate the NMI metrics
(NMI function found in file infoMeasures.R)

Mixing of data series (low value is better)

```
NMIe=NMI(clusters, dataseries[subset])
```

Separation of phenotypes (high value is better)

```
phenotypes=scan("phenotypes.txt", what="character")
NMIp=NMI(clusters, phenotypes)
```

The parameter VAR for the CancerMap function specifies what selection of genes is used (default 15% most variable). Different values can be tested to improve the result.

3) Remapping new samples to an existing t-SNE map. This code is only applicable if t-SNE was used for dimensionality reduction.

```
remapped_coord = run_remapping(originalData, map_coord, newData)
```

4) Plotting clusters and any color vectors on the 2D visualization.

```
Plot_cancermap_clusters(X, peaks, CLUSTER_CENTRE, NAME, SIZE, VAR, BW, TITLE, PATH_OUTPUT)

Plot_color_vector(X, NAME, SIZE, color, TITLE, PATH_OUTPUT, peaks)
```

System

The sample code has been tested with R version 3.3.3 and Bioconductor version 3.2.

Use Case 1: Comparing data-driven sample stratifications with respect to biological vs technical variation using NMI metrics

Description

In this example, the metrics NMIe and NMIp (Mehtonen and Pölönen et al.) are computed for t-SNE maps generated from several AML datasets that represent different experiments (dataseries from NCBI GEO). Meanshift clustering is then used to assign each sample to a cluster. Calculating the NMI metrics can guide parameter selection to mitigate effects of technical variation while preserving separation of biologically relevant disease subtypes.

Usage

The provided R wrapper script computes NMIe and NMIp using a user-specified percentage of variable genes or number of principal components (PCs) (Evaluate_cancermap_NMI.R) and visualizes these metrics in two line plots.

Example analysis, use case 1:

```
# Load gene expression data and annotations.
load("DATA/useCase1/HEMAP_data.Rdata") # 'data' variable.
anno =
read.delim("DATA/useCase1/USE_final_anno_columnIDsorted_data9544_withColorClass.txt", stringsAsFactors = F)

# Subset AML
data <- t(matrix[,anno$colorClass=="AML"])
anno <- anno[anno$colorClass=="AML",]

# Use helper function to simplify GSE and phenotype information in the annotation
source("useCase1/getAMLannotation.R")
aml_anno <- getAMLannotation(anno)
```

Create new output directory for results, if it doesn't already exist.
By default creates the output directory under current working directory.

```
PATH_OUTPUT="output_useCase1/"
dir.create(file.path(getwd(), PATH_OUTPUT), showWarnings = FALSE, recursive=T)
```

Step 1: Calculate NMI for clusters based on phenotype and sample series

Load helper functions

```
source("useCase1/infoMeasures.R") # NMI calculation functions
source("useCase1/calcMeasures.R") # Function to automate NMI calculation
```

Parallelize

```
library(doParallel)
nCores <- min(detectCores(), 20)
registerDoParallel(cores = nCores)
```

Bandwidth parameter for mean-shift clustering.

```
h = 1.5
```

Run calcMeasures with gene selection and with PCA

```
res.gs <- calcMeasures(data = data, phenotype = aml_anno$Cytogenetics, sample_series =
aml_anno$GSE.identifier..experiment., PCA = F)
res.pca <- calcMeasures(data = data, phenotype = aml_anno$Cytogenetics, sample_series =
aml_anno$GSE.identifier..experiment., PCA = T)
```

Step 2: Plot NMI measures

Load library for plots

```
library(ggplot2)
```

Generate plots

```
p.gs <- ggplot(res.gs, aes(x = i, y = value, color = variable)) + geom_line() +
scale_colour_manual(values=c("blue", "red"))
p.gs <- p.gs + labs(title = "Gene selection", x = "% genes", y = "NMI")
p.pca <- ggplot(res.pca, aes(x = i, y = value, color = variable)) + geom_line() +
scale_colour_manual(values=c("blue", "red"))
p.pca <- p.pca + labs(title = "PCA", x = "PCs", y = "NMI")
```

Print to PDF

```
pdf(file.path(PATH_OUTPUT, " NMI_results.pdf "))
plot(p.pgs)
plot(p.pca)
graphics.off()
```

Output
NMI_results.pdf

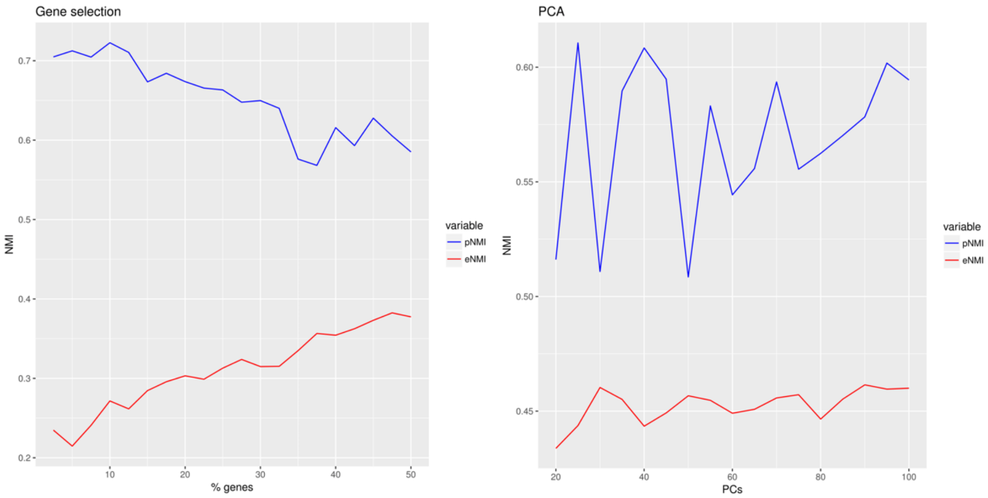

Figure 1. NMI measures plotted when selecting portion of most variable genes (left) and number of principal components (right). Note: Here we run the analysis only with one random generator seed. For actual analysis, we recommend representing the average from multiple runs with different seeds.

Use Case 2: Adding new samples to an existing t-SNE map

Description

This example uses the remapping algorithm we developed to add additional samples to an existing t-SNE map. As a benchmark, 108 samples from GSE49032 dataset are added to Hemap pre-B-ALL t-SNE map.

Usage

The provided R wrapper script (Remapping2Cancermap.R) measures pairwise similarities between the original data samples (ALL t-SNE-map) and new samples and reports position for the new samples in original coordinate space. As validation, the obtained sample coordinates for remapped samples are plotted on the ALL t-SNE map colored by cytogenetic subtype.

Example analysis, use case 2:

Step 1: Load input data and set parameters. The gene expression matrix and t-SNE map coordinates are loaded for the existing ALL t-SNE map. Also, gene expression matrix for new samples is loaded.

Step 2: Remap 108 additional samples to existing t-SNE map. As an additional input, the gene list for top 15% variable genes used for the exiting map are used for filtering the new dataset.

```
remapped_coord = run_remapping(originalData = data, map_coord = coord, newData = newData,
features = top15var_genes)
```

Step 3: Plot new samples on the existing t-SNE map. Coloring the cytogenetic subtype of the remapped samples is used to check placement to correct clusters.

```
plot.remapped(coord_A = coord_orgMap, coord_B = coord_remapped, COLOR_A=col2show,
COLOR_B=col2show_remapped, PATH_OUTPUT = PATH_OUTPUT, NAME = NAME)
```

**Output**
Remapped_samples_singlepage.pdf

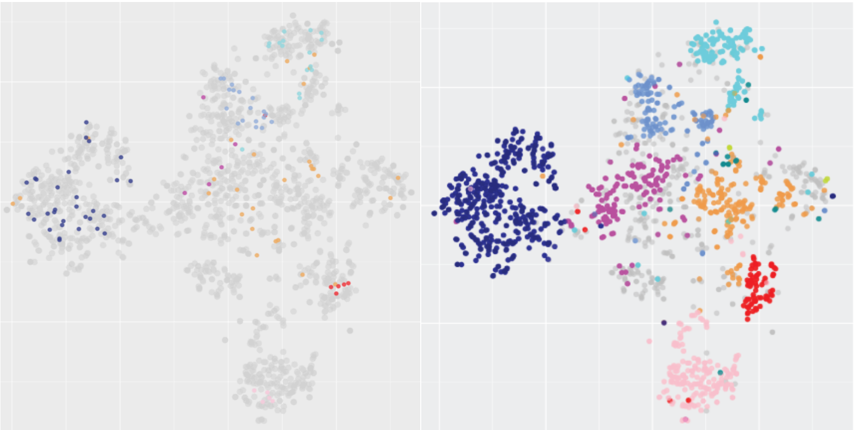

Figure 2. Rscript step 3. a. Remapped samples (left, colors indicate different cytogenetic group) can be compared to annotated cytogenetic types in Hemap ALL t-SNE map (right, indicated in color).

Use Case 3: Comparing an independent dataset using cluster-matching and gene set analysis

Description

In this example, the Ross acute lymphoblastic leukemia (referred to as Ross ALL) study will be jointly analyzed with Hemap ALL samples.

First, a t-SNE map is generated using the Ross ALL samples. In this case, mean-shift clustering using bandwidth parameter 0.9 results in 9 clusters with high correspondence to cytogenetic annotations.

Next, the similar sample groups will be identified from the Hemap ALL sample collection based on gene set scores. For this purpose, genes that correlate with the cluster assignment will be identified, followed by visualization of the gene set scores on the map.

Further statistical analysis can then be used to characterize these sample groups by correlating the cluster assignment with gene set, pathway and annotation features (refer to Mehtonen and Pölönen et al. for examples).

Usage

The provided R-script (Dataset_cancermap_comparison.R) generates a t-SNE map from a new dataset (see input data format below) and from Hemap ALL samples. The gene set scoring is performed using functions implemented in the R/Bioconductor package GSVA.

Example analysis, use case 3:

Load data, set parameters and load libraries. Installs missing R packages automatically.

```
source("useCase3/load_scripts_set_parameters.R")
```

Modify accordingly or use ROSS and Hemap ALL example data

```
source("useCase3/load_data_useCase3.R")
```

Step 1: Generate t-SNE maps and cluster samples on the map using the mean-shift algorithm.

new data t-SNE map

```
clust=CancerMap(data = t(newdata), name = NAME, VAR = VAR, BW = BW, PATH_OUTPUT = PATH_OUTPUT)
```

Hemap t-SNE map

```
clust_hemap=CancerMap(data = t(matrix), name = HEMAP, BW = BW_HEMAP, PATH_OUTPUT = PATH_OUTPUT)
```

Output
cancermap_Ross_rma_u133a_15pct_genes_BH-SNE_mean-shift_BW0.9.txt
cancermap_Ross_rma_u133a_15pct_genes_BH-SNE_mean-shift_BW0.9_cluster_centroids.txt
cancermap_HEMAP_ALL_15pct_genes_BH-SNE_mean-shift_BW1.5.txt
cancermap_HEMAP_ALL_15pct_genes_BH-SNE_mean-shift_BW_cluster_centroids.txt

Read in new t-SNE map coordinate data

```
X=read.delim(paste0(PATH_OUTPUT, "cancermap_", NAME, "_", VAR, "pct_genes_", "BH-SNE_mean-shift_BW", BW, ".txt"), header=T, stringsAsFactors=F)
```

Read in new t-SNE map cluster centroid data

```
peaks=read.delim(paste0(PATH_OUTPUT, "cancermap_", NAME, "_", VAR, "pct_genes_", "BH-SNE_mean-shift_BW", BW, "_cluster_centroids.txt"), header=T, stringsAsFactors=F)
```

Plot clusters with different colors

```
Plot_cancermap_clusters(X, peaks, CLUSTER_CENTRE, NAME, SIZE, VAR, BW, NAME, PATH_OUTPUT)
```

Plot color vector

```
Plot_color_vector(X, NAME, SIZE, newdata_color_vector, NAME, PATH_OUTPUT, peaks)
```

Output
cancermap_Ross_rma_u133a_15pct_genes_BH-SNE_mean-shift_BW0.9_singlepage.pdf
Ross_rma_u133a_singlepage.pdf

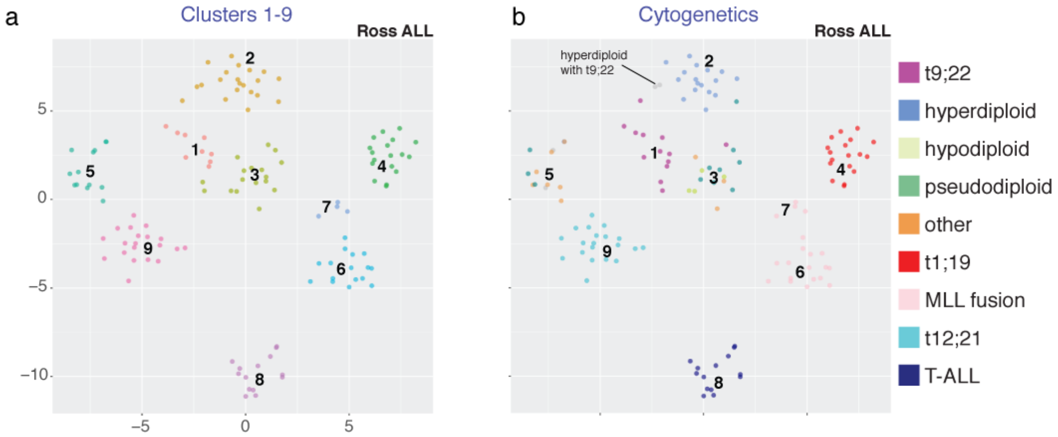

Figure 3. Rscript step 1. a. The data-driven cluster assignment (left, colors indicate different clusters) can be compared to annotated cytogenetic types (indicated in color in b) and correlated with sample molecular features.

```
# Step 2: make gene sets that are identified from Ross TSNE map, run GSVA.

# Obtain cluster number vector
clusters=unique(X[,4])

# Make gene sets to identify similar clusters from Hemap
genesets=unlist(lapply(clusters, Find_correlated_genes, newdata, X), recursive=F)

# Run GSVA to get gene set scores
gsva_es <- gsva(as.matrix(matrix), method="gsva", genesets, mx.diff=F, tau=0.25, verbose=T,
rnaseq=F, min.sz=5, max.sz=500)

# Obtain GSVA score matrix
gsva_es=gsva_es$es.obs
feats=rownames(gsva_es)

# Read in t-SNE map coordinate data
X_hemap=read.delim(paste0(PATH_OUTPUT, "cancermap_", HEMAP, "BH-SNE_mean-shift_BW",
BW_HEMAP, ".txt"), header=T, stringsAsFactors=F)

# Read in t-SNE map cluster centroid data
peaks_hemap=read.delim(paste0(PATH_OUTPUT, "cancermap_", HEMAP, "BH-SNE_mean-
shift_BW", BW_HEMAP, "_cluster_centroids.txt"), header=T, stringsAsFactors=F)

 # Generate plots for each cluster with GSVA score colored
p.all=lapply(feats, Plot_GSVA_scores, gsva_es, VALUE, SIZE, CLUSTER_CENTRE, X_hemap,
peaks_hemap)
```

Make an A4 size figure with multiple panels
```
ggsave(paste0(PATH_OUTPUT, HEMAP, "_", NAME, "_", PATHW, "_multipage.pdf"),
                        do.call(marrangeGrob, list(grobs=p.all, nrow=4, ncol=3)), width = 210, height =
297, units = "mm", dpi=150)
```

Output
HEMAP_ALL_Ross_rma_u133a_clusters_top20_GSVA.Rdata
HEMAP_ALL_Ross_rma_u133a_clusters_top20_multipage.pdf

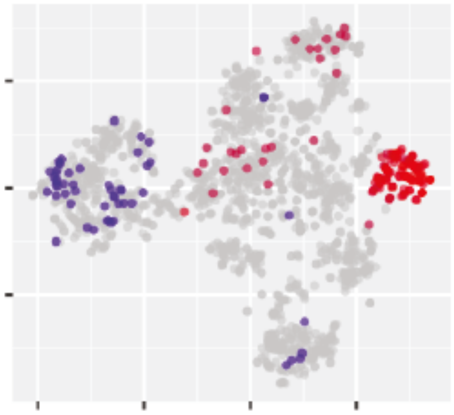

Figure 4. The cluster-specific gene sets distinguish similar samples from Hemap ALL cancer map. Ross cluster 5 gene set score is shown as an example. Red color corresponds to GSVA score above 0.4 and blue below 0.4.

Step 3: Plot cytogenetics in hemap TSNE map.

```
Plot_color_vector(X_hemap, HEMAP, SIZE, hemap_color_vector, HEMAP, PATH_OUTPUT,
peaks_hemap)
```

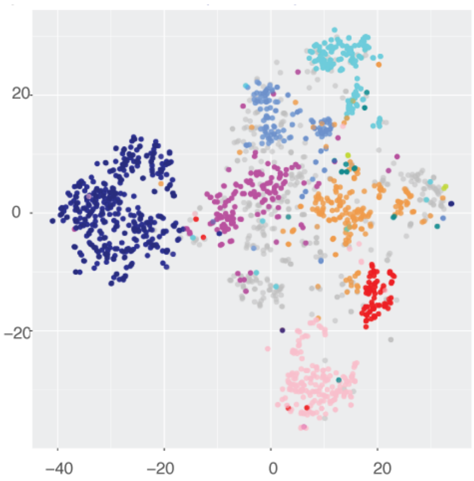

Figure 5. The annotated cytogenetic sample type is colored as in Figure 3. The correspondence of gene set score based matching (in Figure 3) with the annotated sample category can be evaluated.

Input data requirements

This section describes what is needed to run similar analysis for any new dataset.

1) The new dataset to be compared should be pre-processed as similarly as possible to the reference dataset. The following format is expected for script inputs:

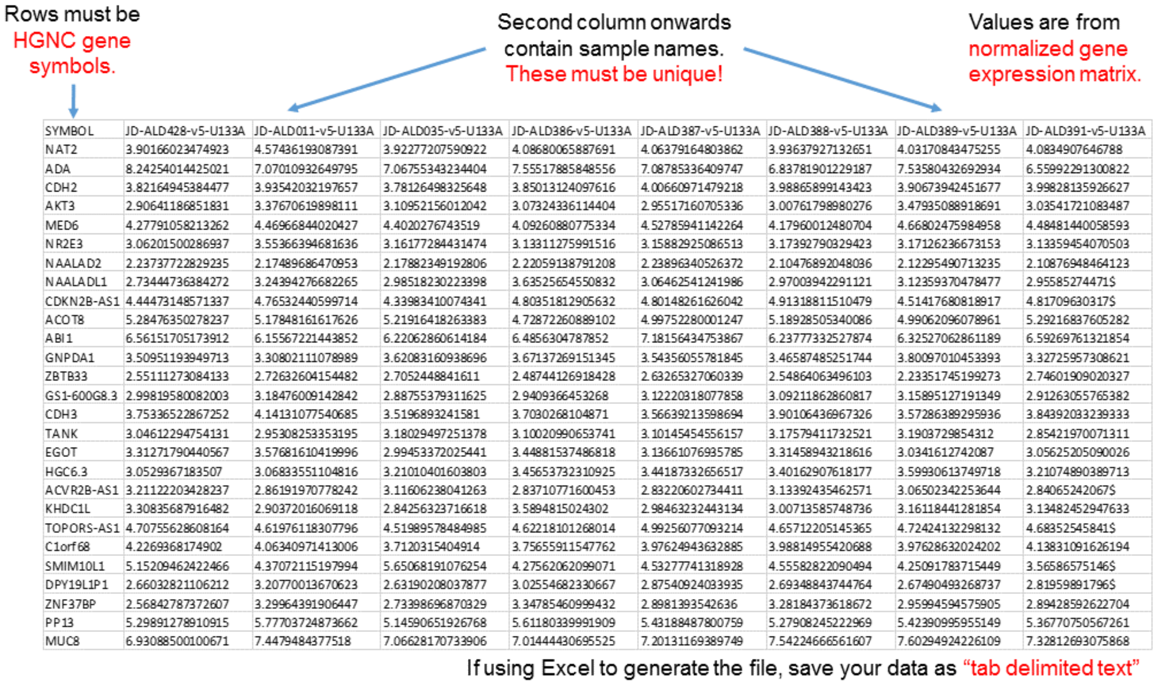

SYMBOL	JD-ALD428-v5-U133A	JD-ALD011-v5-U133A	JD-ALD035-v5-U133A	JD-ALD386-v5-U133A	JD-ALD387-v5-U133A	JD-ALD388-v5-U133A	JD-ALD389-v5-U133A	JD-ALD391-v5-U133A
NAT2	3.90166023474923	4.57436193087391	3.92277207590922	4.08680065887691	4.06379164803862	3.93637927132651	4.03170843475255	4.0834907646788
ADA	8.24254014425021	7.07010932649795	7.06755343234404	7.55517885848556	7.08785336409747	6.83781901229187	7.53580432692934	6.55992291300822
CDH2	3.82164945384477	3.93542032197657	3.78126498325648	3.85013124097616	4.00660971479218	3.98865899143423	3.90673942451677	3.99828135926627
AKT3	2.90641186851831	3.37670619898111	3.10952156012042	3.07324336114404	2.95517160705336	3.00761798980276	3.47935088918691	3.03541721083487
MED6	4.27791058213262	4.46966844020427	4.4020276743519	4.09260880775334	4.52785941142264	4.17960012480704	4.66802475984958	4.48481440058593
NR2E3	3.06201500286937	3.55366394681636	3.16177284431474	3.13311275991516	3.15882925086513	3.17392790329423	3.17126236673153	3.13359454070503
NAALAD2	2.23737722829235	2.17489686470953	2.17882349192806	2.22059138791208	2.23896340526372	2.10476892048036	2.12295490713235	2.10876948464123
NAALADL1	2.73444736384272	3.24394276682265	2.98518230223398	3.63525654550832	3.06462541241986	2.97003942291121	3.12359370478477	2.95585274471$
CDKN2B-AS1	4.44473148571337	4.76532440599714	4.33983410074341	4.80351812905632	4.80148261626042	4.91318811510479	4.51417680818917	4.81709630317$
ACOT8	5.28476350278237	5.17848161617626	5.21916418263383	4.72872260889102	4.99752280001247	5.18928505340086	4.99062096078961	5.29216837605282
ABI1	6.56151705173912	6.15567221443852	6.22062860614184	6.4856304787852	7.18156434753867	6.23777332527874	6.32527062861189	6.59269761321854
GNPDA1	3.50951193949713	3.30802111078989	3.62083160938696	3.67137269151345	3.54356055781845	3.46587485251744	3.80097010453393	3.32725957308621
ZBTB33	2.55111273084133	2.72632604154482	2.7052448841611	2.48744126918428	2.63265327060339	2.54864063496103	2.23351745199273	2.74601909020327
GS1-600G8.3	2.99819580082003	3.18476009142842	2.88755379311625	2.9409366453268	3.12220318077858	3.09211862860817	3.15895127191349	2.91263055765382
CDH3	3.75336522867252	4.14131077540685	3.5196893241581	3.7030268104871	3.56639213598694	3.90106436967326	3.57286389295936	3.84392033239333
TANK	3.04612294754131	2.95308253353195	3.18029497251378	3.10020990653741	3.10145454556157	3.17579411732521	3.1903729854312	2.85421970071311
EGOT	3.31271790440567	3.57681610419996	2.99453372025441	3.44881537486818	3.13661076935785	3.31458943218616	3.0341612742087	3.05625205090026
HGC6.3	3.0529367183507	3.06833551104816	3.21010401603803	3.45653732310925	3.44187332656517	3.40162907618177	3.59930613749718	3.21074890389713
ACVR2B-AS1	3.21122203428237	2.86191970778242	3.11606238041263	2.83710771600453	2.83220602734411	3.13392435462571	3.06502342253644	2.84065242067$
KHDC1L	3.30835687916482	2.90372016069118	2.84256323716618	3.5894815024302	2.98463232443134	3.00713585748736	3.16118441281854	3.13482452947633
TOPORS-AS1	4.70755628608164	4.61976118307796	4.51989578484985	4.62218101268014	4.99256077093214	4.65712205145365	4.72424132298132	4.68352545841$
C1orf68	4.2269368174902	4.06340971413006	3.7120315404914	3.75655911547762	3.97624943632885	3.98814955420688	3.97628632024202	4.13831091626194
SMIM10L1	5.15209462422466	4.37072115197994	5.65068191076254	4.27562062099071	4.53277741318928	4.55582822090494	4.25091783715449	3.56586575146$
DPY19L1P1	2.66032821106212	3.20770013670623	2.63190208037877	3.02554682330667	2.87540924033935	2.69348843744764	2.67490493268737	2.81959891796$
ZNF37BP	2.56842787372607	3.29964391906447	2.73398696870329	3.34785460999432	2.8981393542636	3.28184373618672	2.95994594575905	2.89428592622704
PP13	5.29891278910915	5.77703724873662	5.14590651926768	5.61180339991909	5.43188487800759	5.27908245222969	5.42390995955149	5.36770750567261
MUC8	6.93088500100671	7.4479484377518	7.06628170733906	7.01444430695525	7.20131169389749	7.54224666561607	7.60294924226109	7.32812693075868

2) Clinical classification or established molecular subtypes for the disease in question are required at least for some samples to verify and optimize the obtained lower-dimensional representation of the sample clusters, and to validate remapping results.

```
sample1    MLL
sample2    other
sample3    MLL
```

subtype=c("MLL", "other", "MLL")

3) Different sample features (cytogenetic type, clinical features, mutation data, etc) can be colored on the 2D visualizations (t-SNE maps). Use the sample annotations available from your study to create vectors that specify the colors to be used in the visualization. You may find e.g. this page helpful
http://www.stat.columbia.edu/~tzheng/files/Rcolor.pdf

```
sample1    MLL
sample2    other
sample3    MLL
```

Your color vector could be specified
myColors=c("pink", "orange", "pink")

Parameters

Cancermap.R

data	Data matrix where rows represent samples and columns represent features.
name	Parameter used in the naming of the output files.
VAR	Percentage of most variable features to retain in the data matrix for t-SNE.
BW	Bandwidth parameter for mean-shfit clustering. Lower the bandwidth, more dense the clusters found.
PATH_OUTPUT	Path where to save the output files.

run_remapping.R

originalData	Gene expression data from original map.
map_coord	Coordinates from originalData TSNE projection.
newData	Gene expression data from new samples.
perplexity	"Soft" number of neighbors assumed. Used in TSNE run.
theta	Portion of approximation used when running Barnes-Hut algorithm.

Plot_cancermap_clusters.R

X	"Coordinate file" of a generated cancer map, where 1st column is the ID a sample, 2nd and 3rd column are the x and y coordinates respectively, and 4th column is the cluster label.
peaks	Contains the cluster centroid coordinates.
CLUSTER_CENTRE	Boolean variable whether to plot cluster centroids to cancer maps.
NAME	Name to be used on the output file.
SIZE	Variable defining point size in cancer maps.
VAR	Percentage of most variable genes included when generating X. Used in naming the output file.
BW	Bandwidth parameter for mean-shift clustering. Used in naming the output file.
TITLE	Title for the plot.
PATH_OUTPUT	Path where to write output files.

Plot_color_vector.R

X	"Coordinate file" of a generated cancer map, where 1st column is the ID a sample, 2nd and 3rd column are the x and y coordinates respectively, and 4th column is the cluster label.
NAME	Name to be used on the output file.
SIZE	Variable defining point size in cancer maps.
color	User-specified color vector. Same length as number or samples in X.
TITLE	Title for the plot.
PATH_OUTPUT	Path where to write output files.
